# Chitosan Grafted Adsorbents for Diclofenac Pharmaceutical Compound Removal from Single-Component Aqueous Solutions and Mixtures

**DOI:** 10.3390/polym11030497

**Published:** 2019-03-14

**Authors:** Areti Tzereme, Evi Christodoulou, George Z. Kyzas, Margaritis Kostoglou, Dimitrios N. Bikiaris, Dimitra A. Lambropoulou

**Affiliations:** 1 Laboratory of Environmental Pollution Control, Department of Chemistry, Aristotle University of Thessaloniki, GR–541 24 Thessaloniki, Greece; areti_tz_1901@windowslive.com; 2 Laboratory of Polymer Chemistry and Technology, Department of Chemistry, Aristotle University of Thessaloniki, GR-541 24 Thessaloniki, Greece; evicius@gmail.com (E.C.); dbic@chem.auth.gr (D.N.B.); 3 Hephaestus Advanced Laboratory, Eastern Macedonia and Thrace Institute of Technology, GR-654 04 Kavala, Greece; kyzas@teiemt.gr; 4 Laboratory of General and Inorganic Chemical Technology, Department of Chemistry, Aristotle University of Thessaloniki, GR-541 24 Thessaloniki, Greece; kostoglu@chem.auth.gr

**Keywords:** chitosan, adsorption, pharmaceuticals, diclofenac, wastewaters

## Abstract

The main purpose of this study was to investigate the synthesis of some cross-linked carboxyl-grafted chitosan derivatives to be used as selective adsorbents for diclofenac (DCF) pharmaceutical compounds from aqueous mixtures. Four different materials were synthesized using succinic anhydride (CsSUC), maleic anhydride (CsMAL), itaconic acid (CsITA), and *trans*-aconitic acid (CsTACON) as grafting agents. After synthesis, scanning electron microscopy (SEM), Fourier-transform infrared spectroscopy (FTIR), and X-ray diffraction (XRD) were performed before and after DCF adsorption. In addition, a complete adsorption evaluation was carried out for all materials studying some important parameters. The optimum pH was 4; the amino groups of DCF can be protonated at pH = 4 (–NH^+^), so this groups can easily attract the clear negatively carboxyl moieties (–COO^−^) of the chitosan adsorbents. The *Q*_m_ for CsTACON was higher than those of the other materials, at all temperatures studied. By altering the temperature from 25 to 35 °C, an increase (16%) of *Q*_m_ (from 84.56 to 98.34 mg g^−1^) was noted, while similar behavior was revealed after a further increase of temperature from 35 to 45 °C, improving by 5% (from 98.34 to 102.75 mg g^−1^). All isotherms were fitted to Langmuir, Freundlich, and Langmuir-Freundlich (L-F) models). In addition, a kinetic model was proposed taking into account not only the interactions but also the diffusivity of the molecule (DCF) into the polymeric network. The behavior of the prepared chitosan materials in simultaneously removing other compounds (synergetic or antagonistic) was also evaluated by experiments performed in mixtures. DCF presented the highest removal from the mixture in the order: CsTACON (92.8%) > CsITA (89.5%) > CsSUC (80.9%) > CsMAL (66.2%) compared to other pharmaceutical compounds (salicylic acid, ibuprofen and ketoprofen). Desorption was achieved by using different eluants (either water or organic). The highest desorption ability was found for acetone (100% for CsTACON, CsSUC, CsMAL and 77% for CsITA) for all materials.

## 1. Introduction

Numerous recent studies have already been published attempting to approach and overcome the crucial problem of drugs, pharmaceuticals and pharmaceuticals and personal care products (PPCPs) in aqueous media. Nowadays, the total amount of pharmaceutical compounds increases [1]. Furthermore, there are also drug compounds which are excreted from the human body without any significant change to their structure and can be also traced in urban wastewaters. The hydrophilic character of the latter, as well as their low removal at wastewater treatment plants (WWTPs), render them precarious compounds that, through the water cycle, can finally reach the drinking water [2,3].

The possible adverse environmental impact of pharmaceutically active compounds and their fate in the aquatic environment is a critical issue that raises concerns nowadays [3,4,5]. Diclofenac (DCF), a common non-steroidal anti-inflammatory drug with analgesic use, is such an example with an ever-increasing consumption, followed by a rather inappropriate disposal and incomplete removal in WWTPs. That leads to notably high levels of the drug in final wastewater effluents [6,7,8]. DCF has been classified as a priority for thorough investigation due to its risk perception, and thus it has recently entered the so-called “watch list” of similar priority substances under the Water Framework Directive [9]. Its global consumption is currently estimated at 940 tons per year, with a defined daily dose of 100 mg [10,11].

Once the traditional water and wastewater treatment technologies proved ineffective to successfully remove several compounds, there is an ongoing discussion about alternative treatment methods that will be able to eliminate them. Several works on the removal of antibiotics from aqueous solutions have been recently published [12,13]. The removal procedure of pharmaceuticals is already well-reported and is usually based on photocatalytic degradation [14,15,16], ozonation [17,18] or adsorption onto various materials such as activated carbon or carrageenans [19,20]. Among them, adsorption appears to be quite promising, and perhaps the most convenient, technique that can easily fit the current water treatment processes [20].

A very promising adsorbent material, which recently is widely used, is chitosan [21,22,23,24,25]. Chitosan (poly-β-(1 → 4)-2-amino-2-deoxy-d-glucose) has the advantage of easy modification (grafting with various functional groups), so it can be easily transformed to the appropriate adsorbent each time. Some of its unique properties such as biocompatibility, biodegradability and non-toxicity, along with its adsorption ability, turn chitosan into a very promising and eco-friendly adsorbent material [26,27,28,29,30], which can be also combined with agro-wastes (coffee) for pharmaceutical removal [31]. Our research team has been extensively studying the use of chitosan in adsorption applications (i.e., for environmental pollutants such as dyes [32,33,34,35,36], heavy metals [37,38,39,40,41,42], and drugs [43]). This long-time experience, combined with the abundant literature that reports the impressive efficiency of chitosan as an adsorbent [44,45,46,47], led us to choose it for this particular study.

More specifically, the modification of chitosan was performed by grafting carboxylic groups of different compounds onto the chitosan backbone. Four different cross-linked materials were synthesized in total, using succinic anhydride, maleic anhydride, itaconic acid, and *trans*-aconitic acid as grafting agents. Diclofenac (DCF) was selected as the target pollutant, because of its ubiquity in aqueous media as mentioned above. Moreover, special attention is given recently to the removal of DCF from water with various methods (adsorption, oxidation, photocatalysis) [48,49,50,51,52]. DCF has several reactive groups like –Cl, –COOH and >NH, which can interact with chitosan’s >NH_2_ and –OH groups. In order to increase its efficiency (CS) to interact with DCF, carboxyl derivatives have been prepared, since it is well known that –COOH groups can form hydrogen bonds with >NH groups. The aim was to compare and check which type of those carboxyl groups grafted in CS can better remove DCF pollutant from aqueous solutions. It was decided to test the prepared grafted chitosan materials and not just the neat chitosan because the scope of this study was to investigate which type of grafting will be ideal and not simply check if grafting will increase the DCF removal. Following a detailed characterization of the prepared materials using different techniques (such as scanning electron microscopy (SEM), Fourier-transform infrared spectroscopy (FTIR), X-ray diffraction (XRD)), the adsorption evaluation was performed. Factors that considerably affect adsorption were also investigated, namely the effect of pH, initial drug concentration, and temperature. To examine the behavior of chitosan materials in regard to the simultaneous removal of two or more different compounds (synergetic or antagonistic), experiments were carried out in mixtures as well. In addition, a kinetic model was proposed based on theory and the resultant experimental points.

## 2. Materials and Methods

### 2.1. Materials and Reagents

Commercial chitosan (CS) powder of high molecular weight (mol wt 310–375 kDa, >75% deacetylated, Sigma-Aldrich), succinic anhydride ≥99.0% (SUC), maleic anhydride ≥99.0% (MAL), itaconic acid ≥99.0% (ITA), and *trans*-aconitic acid 98.0% (TACON), ammonium persulfate ≥98.0% (APS), were purchased from Sigma-Aldrich (St. Louis, MO, USA). The water used for the adsorption experiments was purified using a Barnstead water purification system (Dubuque, IA, USA). Diclofenac (DCF), salicylic acid (SAC), ibuprofen (IBF) and ketoprofen (KPF), with assays ≥99.9%, were kindly provided by Pharmathen SA (Athens, Greece).

### 2.2. Pharmaceutical Compounds as Model Pollutants

**Figure 1:**
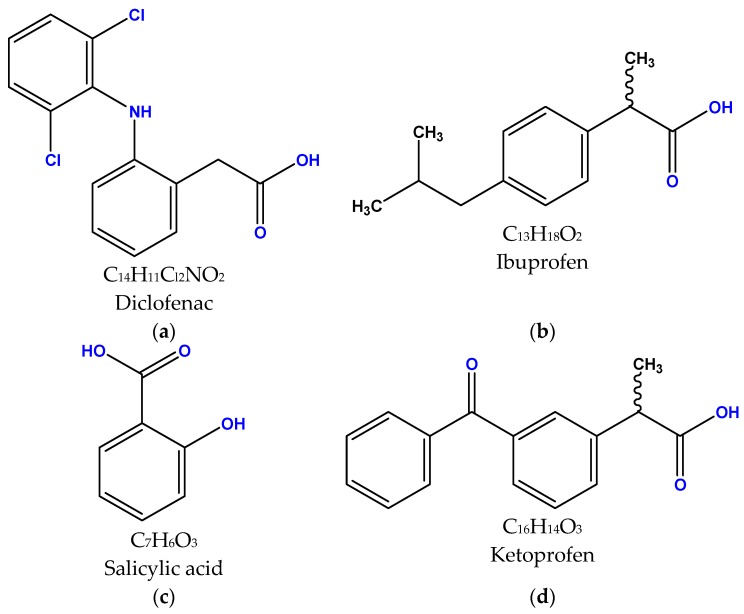
Molecular formula and chemical structures of the used pharmaceutical compounds: (**a**) diclofenac, (**b**) ibuprofen, (**c**) salicylic acid and (**d**) ketoprofen.

For the single-component adsorption experiments, diclofenac was used to examine the adsorption ability of synthesized chitosan derivatives. Then, in order to check the adsorption behavior of chitosan in mixtures consisted of more than one drug compound, three more pharmaceutical compounds were also added to the solution: salicylic acid, ibuprofen and ketoprofen. The above compounds were selected because they belong to the same category of pharmaceuticals (anti-inflammatory/analgesic properties). The structures and molecular formulas are given in Figure 1.

### 2.3. Synthesis of Grafted Chitosan Adsorbents

The synthesis yields (%) were relatively high (~60%–90%) for all materials; 89.7, 74.2, 66.3, and 80.1% for CsSUC, CsITA, CsMAL and CsTACON, respectively.

#### 2.3.1. Chitosan Grafted with Succinic Anhydride (CsSUC)

The synthesis of CsSUC was mostly set up according to the Hirano and Moriyasu method [53], after modifying some ratios [54]. In brief, 3 g of high molecular weight chitosan was dissolved in an aqueous acetic acid solution (6% *v*/*v*). The solution was diluted with 300 mL of methanol and left under constant stirring for 1 h. Succinic anhydride (4 g) was subsequently dissolved in 100 mL of acetone and the solution was slowly transferred into the above-mentioned chitosan solution for 30 min at 25 °C. Then, the reaction was allowed to take place for 18 h, at room temperature. The obtained viscous solution was afterwards diluted with 400 mL of deionized water and the pH was adjusted to 10 (using 2 mol L^−1^ NaOH solution). The resulting product was well washed in a mixture of water/acetone (1:1) and the solution was freeze-dried, after dialysis for 3 days, obtaining a cotton-white material (CsSUC).

#### 2.3.2. Chitosan Grafted with Maleic Anhydride (CsMAL)

The synthesis of CsMAL was performed in a similar way, according to our previous studies [54], after modifying some ratios. High molecular weight chitosan (3.2 g) was dissolved in 250 mL aqueous acetic acid solution (2% *v*/*v*) and 125 mL methanol was also added. To graft with functional groups, 8 g of maleic anhydride was dissolved in 150 mL of acetone and the solution was added to the chitosan solution prepared above at 50 °C. The mixture was mechanically stirred overnight at room temperature. The resulting solution was diluted with 500 mL of 0.5 mol L^−1^ NaOH and 100 mL ethanol. The produced material was then filtrated and freeze-dried after dialysis for 3 days.

#### 2.3.3. Chitosan Grafted with Itaconic Acid (CsITA)

The graft copolymerization of itaconic acid on chitosan was carried out [55] in a two-necked round-bottomed glass reactor. In a typical grafting method, 2 g of CS were dissolved in 200 mL acetic acid (1% *v*/*v*) and APS solution (6.58 × 10^−5^ mol, 25 mL) were transferred into the reactor and stirred magnetically at 100 rpm for 10 min. Then, 0.005 mol of IA that was dissolved in a methanol solution (MeOH) was added dropwise. The grafting reaction was carried out at 60 °C for 4 h under a nitrogen atmosphere. Then, the chitosan derivative was removed and washed several times with purified water. The powder was dried under reduced pressure at 60 °C to obtain the final product of CsITA, which was purified using a Soxhlet apparatus and methanol to remove the unreacted IA.

#### 2.3.4. Chitosan Grafted with Trans-Aconitic Acid (CsTACON)

For the preparation of *trans*-aconitic acid-modified chitosan (CsTACON) [56], 1.57 g *trans*-aconitic acid was dissolved in a MeOH solution of EDC (1.7 g EDC per 50 mL MeOH) and then gradually added into a chitosan solution (1.5 g chitosan in 150 mL acetic acid (1% *v*/*v*)). The amount of *trans*-aconitic acid used, corresponded to 2:1 molar ratio of reactive groups COOH/NH_2_. The reaction continued overnight at room temperature and under constant mechanical stirring. A solution of 2 mol L^−1^ NaOH was used to adjust the pH of the obtained solution to 10. The filtrated product was washed with deionized water and freeze-dried after 3 days of dialysis. The grafting percentage (GP) was calculated based on the equation GP = (*M*_fin_ − *M*_in_)/*M*_in_ × 100%, where, *M*_in_ and *M*_fin_ are the mass of chitosan before and after grafting process, respectively.

#### 2.3.5. Cross-Linking of Chitosan Derivatives

**Figure 2:**
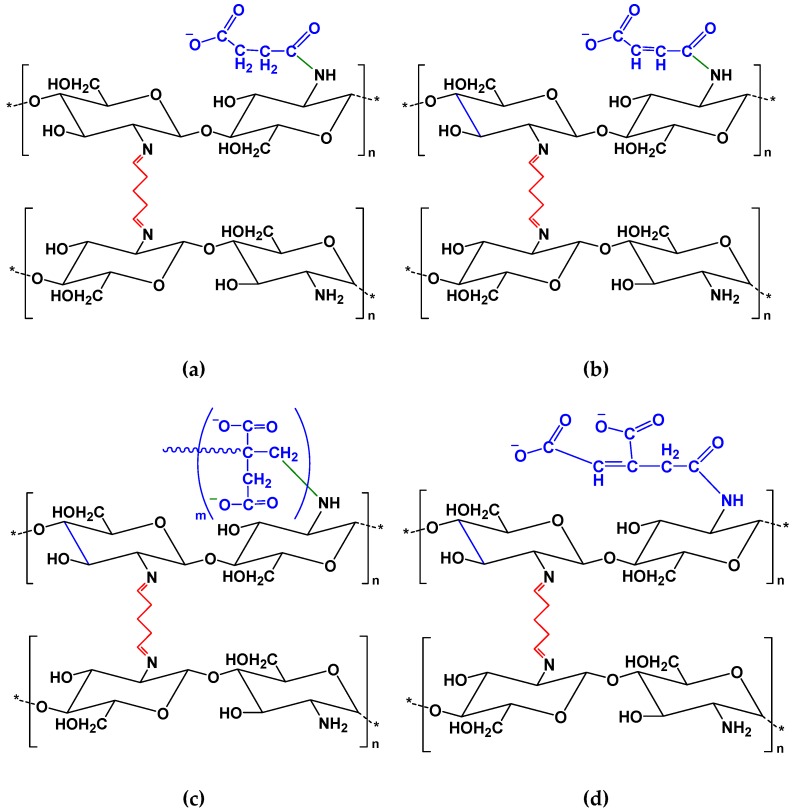
Chemical structure of the prepared chitosan grafted and cross-linked adsorbents: (**a**) CsSUC; (**b**) CsMAL; (**c**) CsITA; (**d**) CsTACON.

Glutaraldehyde (GLA) was used as cross-linking agent for all materials [32,33,35,38,57,58]. The cross-linking resulted after Schiff’s base reaction between aldehyde of the cross-linking agent and amine moieties of chitosan so as to form imine functions. In particular, by gradually adding a solution of each sample (0.5 g of the sample in 50 mL of acetic acid (3% *v*/*v*)) into 50 mL of an aqueous GLA solution (1% *v*/*v*). The reaction was continued under magnetic stirring at 25 °C for 30 min. All cross-linked materials were washed several times with deionized water and further purified using acetone in the Soxhlet apparatus. All of the above cross-linked materials are schematically presented in Figure 2.

### 2.4. Characterization Techniques

The characterization of the prepared grafted chitosans was based on four basic techniques. FTIR was used to examine the functional groups of chitosans after synthesis (before adsorption experiments). The same characterizations were applied after pharmaceuticals experiments to examine the interaction between drug molecules and chitosan groups. The FTIR spectra were taken using a Perkin-Elmer FTIR spectrometer (model Spectrum One, Perkin Elmer, Dresden, Germany). Briefly, 5 mg of each sample was mixed with 180 mg of KBr in mortal. The mixture was pressed under 5 tons for 2 min and the final-for-analysis pellet was formed. This was placed into an attachment in the optical compartment and FTIR spectra were obtained. Infrared (IR) absorbance spectra were taken in the range of 450–4000 cm^−1^ at 4 cm^−1^ resolution with 20 co-added scans. All spectra submitted to baseline correction and normalization to 1. The patterns of X-ray diffraction (XRD) were taken using Rigaku MiniFlex II diffractometer with Bragg-Brentano geometry (θ, 2θ) and Ni-filtered CuKα radiation. The analysis was performed on the synthesized chitosans. The samples were scanned over the internal range of 5–60°, step 0.05°, rate 1° min^−1^. Scanning electron microscopy (SEM) images were performed with an electron microscope (model Zeiss Supra 55 VP, Jena, Germany). The accelerating voltage was 15 kV and the scanning was performed in-situ on a sample powder.

### 2.5. Adsorption Experimental Design

#### 2.5.1. Single-Component DCF Solution

The experimental design of this work mainly includes the adsorption evaluation of chitosan adsorbents for the removal of DCF pharmaceutical compound from aqueous media. To achieve this, the effect of pH was initially studied. The adsorption process was carried out preparing the synthetic drug solutions in conical flasks using particular initial drug concentrations (*C*_i_) diluted in fixed volume of deionized water (*V*); then the flasks were placed to thermostatically-controlled water bath (model Grant Instruments OLS Aqua Pro, Cambridge, UK) with fixed shaking rate (*N*), but with different temperature (*T*). The flasks were kept under shaking for a particular time (*t*). The mass of chitosan (*m*) used for adsorption experiments was kept constant for comparative reasons. The residual drug concentrations (*C*_e_) in the liquid phase (after the end of adsorption) were measured. To clearly present the adsorption experimental conditions, the following Table 1 was given.

#### 2.5.2. Mixtures

The experimental design for the removal of drug compounds from aqueous media is based on the optimum conditions found from single-component experiments. The initial concentration of 30 mg L^−1^ for each drug (SAC, IBF, KPF) was added to 10 mL of deionized water. The pH of the solution was adjusted to the optimum value found (4), as well as all other optimum values (*T* = 25 °C; *t* = 30 min; *N* = 150 rpm; *m* = 0.01 g). Then, the residual concentrations (*C*_e_) of each drug was analyzed.

### 2.6. Desorption Experimental Design

After adsorption experiments (where the chitosan derivatives were exposed to 30 mg L^−1^ of DCF solution at 25 °C (at pH = 4), the samples were collected and filtered, using fixed pore-sized membranes. Desorption experiments were performed by mixing the collected, after adsorption, amount of drug-loaded materials (0.01 g) with 10 mL of different eluent (deionized water at pH = 10; methanol, acetone; CH_2_Cl_2_; CHCl_3_). The evaluation of desorption was realized as desorption percentage, calculated from the difference between the loaded amount of DCF on adsorbent after adsorption and the amount of DCF in solution after desorption. This procedure was realized to determine the optimum desorption eluent.

### 2.7. Chromatographic Analysis

The LC system consisted of a SIL 20A auto-SWE sampler with the volume injection set to 20 μL and LC-20AB pump both from Shimadzu (Kyoto, Japan). Chromatographic separation was achieved using a C_18_ (Athena) analytical column 250 × 4.6 mm with 5 μm particle size. Detection was performed using an SPD 20A DAD detector coupled in series with the LC-MS 2010 EV mass selective detector, equipped with an atmospheric pressure electrospray ionization (ESI) source. The samples were analyzed using the ESI interface in negative mode (NI) for DCF. The mobile phase consisted of water with 0.1% formic acid (A) and methanol (B) in an isocratic elution program (10% A: 90% B). The column temperature was set at 40 °C and the flow rate was 0.4 mL min^−1^. The drying gas was operated at flow 10 L/min at 200 °C. The nebulizing pressure was 100 psi, capillary voltage was 4500 V for positive ionization and −3500 V for negative ionization and the fragmentation voltage was set at 5 V. For each compound the precursor molecular ion in the selected-ion monitoring (SIM) mode was acquired ([M–H] 294 m/z for DCF).

### 2.8. Error Analysis

Determination of the best isotherm model is only possible through analysis of the correlation coefficient (R^2^). Although efficient, this indicator is limited to solving isotherm models that present linear forms. Therefore, in this work, three different error functions were employed in order to discover the isotherm model most suitable for representing the experimental data.

The sum of squared errors (SSE) (Equation (1a)) is the most commonly utilized error function. However, it has the disadvantage of providing isotherm parameters that present better adjustment to the final portion of the isotherm. This is due to the magnitude of the errors, which causes an increase in squared errors as the adsorbate concentration increases. The sum of absolute errors (SAE) (Equation (1b)) also provides better adjustments for higher concentrations. This occurs because an increase in the concentration range causes an increase in error. The average relative error (ARE) (Equation (1c)) function attempts to minimize the fractional error distribution across the entire concentration range.

(1a) $$\mathrm{SSE}={\sum_{i=1}^{n}\left(Q_{e,\mathrm{calc}}-Q_{e,\exp}\right)}_{i}^{2}$$

(1b) $$\mathrm{SAE}=\sum_{i=1}^{n}{\left|Q_{e,\mathrm{calc}}-Q_{e,\exp}\right|}_{i}$$

(1c) $$\mathrm{ARE}=\frac{100}{n}\sum_{i=1}^{n}{\left|\frac{Q_{e,\mathrm{calc}}-Q_{e,\exp}}{Q_{e,\exp}}\right|}_{i}.$$

## 3. Results and Discussion

The presentation of experimental results and the respective discussion is divided into some major sections. At first, the characterization of the prepared uncross-linked chitosan grafted materials, as well as an investigation of the evolved interactions between the cross-linked adsorbents and DCF confirmed by FTIR spectroscopy are given. Then, the adsorption evaluation of chitosan derivatives synthesized is presented based on some key-factors as the pH and temperature (isotherms) of the solution (wastewater), and the contact time (kinetics). In addition, special focus is given on the adsorption of pharmaceuticals existed in a synthetic mixture (consisted of five drug compounds) and the desorption potential of the chitosan materials produced.

### 3.1. Characterizations

The grafting percentage of all monomers into chitosan chains were determined. The grafting percentage (GP) indicates the increase in weight of neat CS subjected to grafting with a monomer. GP values of 44.3, 37.6, 15.4 and 22.6% were calculated for CsSUC, CsITA, CsMAL and CsTACON, respectively.

**Figure 3:**
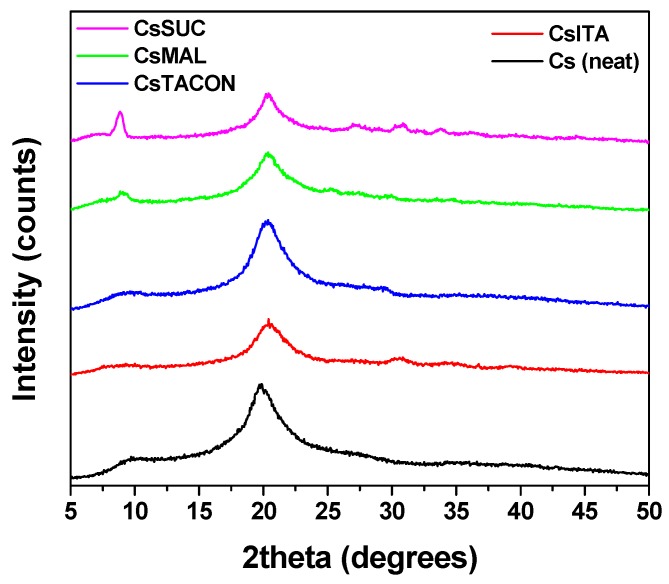
XRD patterns of the synthesized uncross-linked materials.

Regarding the neat CS, XRD diffractograms displayed an amorphous halo with two broad peaks 2θ = 10° and 20°, confirming the semi-crystalline nature of the polymer Figure 3. In the case of the modified derivatives, XRD analysis revealed that all adsorbents are completely amorphous since only the broad peak at approximately 2θ = 20° was recorded. This was expected as the grafting of small molecules on the macromolecular chitosan backbone reduces its folding ability, and consequently the crystal structure formation. Additionally, in the case of two patterns (CsSUC and CsMAL), the second characteristic peak of CS at 2θ = 10° was also recorded. This is probably attributed to the fact that succinic and maleic anhydride monomers are structurally similar and relatively smaller compared to trans-aconitic and itaconic acid, and thus, they don’t significantly affect CS prior crystalline form. XRD analysis showed no significant changes in the structure after cross-linking.

**Figure 4:**
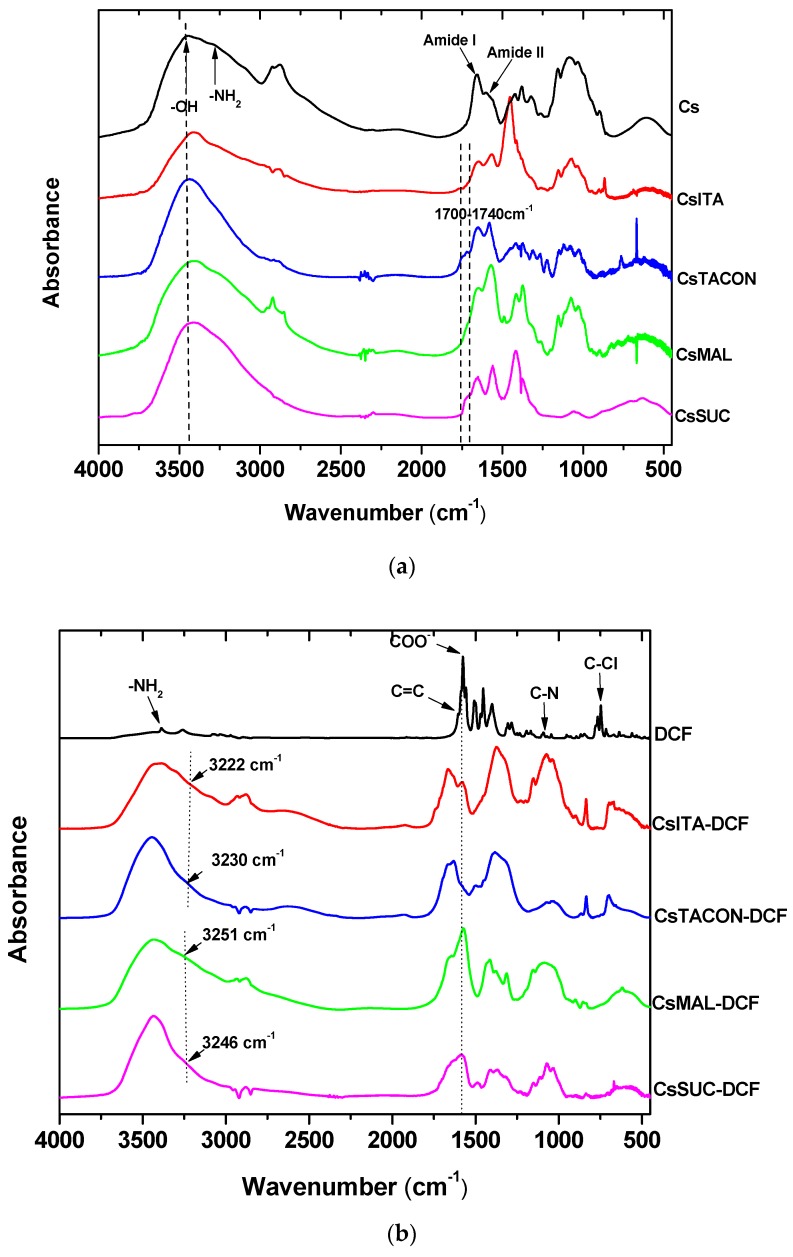
FTIR spectra of the synthesized chitosan derivatives: (**a**) before cross-linking in comparison with neat CS; (**b**) after cross-linking and DCF adsorption.

The structures of the modified chitosan derivatives were confirmed by FTIR spectroscopy. Figure 4a shows the recorded spectra of neat chitosan and the prepared adsorbents before the adsorption of DCF. As can be seen, the main bands of neat CS are located at 3000–3600 cm^−1^ (a broad band attributed to –OH with maximum at 3457 cm^−1^ and a shoulder at 3200–3270 cm^−1^ due to –NH_2_ stretching), 1659 cm^−1^ (>CO stretching-amide I), 1585 cm^−1^ (amide II), 1419 cm^−1^ (C–H and O–H vibrations), 1152 cm^−1^ (anti-symmetric stretching of the C–O–C bridge) and 1078 cm^−1^ (skeletal vibrations involving the C–O stretching), which are characteristics of its polysaccharide structure. In regards to the modified derivatives, most of the characteristic peaks of the neat CS are recorded in their spectra. Such new peaks are recorded in 1700–1740 cm^−1^ due to the addition of carboxylic derivatives in chitosan backbone, a fact that indicates the successful modification of chitosan while the intensity of –NH_2_ band was smaller proving that the reaction took place at the *N*-position. In addition, the band corresponding to –OH stretches became sharper and shifted to lower wavenumbers (from 3457 cm^−1^ to about 3425 cm^−1^) indicating the presence of hydrogen bonding between the introduced carboxylic monomers and the hydroxyl groups of chitosan. In addition, a small shift was recorded in chitosan’s amino groups from 3255–3260 cm^−1^ to about 3240–3250 cm^−1^, proving that these groups can also interact with added carboxyl groups.

In a further step, FTIR spectroscopy was adopted to determine any possible interactions between DCF and the prepared cross-linked adsorbents. The FTIR spectrum of DCF (Figure 4b) confirmed the existence of peaks at 3388, 1604, 1579, 1283, 1043 and 746 cm^−1^ due to NH stretching, C=C stretching, COO– stretching, C–Cl and C–N stretching and C–Cl bending respectively, as characteristic functional groups of the drug.

In regards to the chitosan derivatives after the drug sorption, FTIR analysis showed the characteristic peaks of derivatives and this of DCF at 1579 cm^−1^, which is the strongest. Examining this peak in adsorbents it can be seen that was shifted in all derivatives to slightly lower positions (1570–1575 cm^−1^) indicating that the carboxylic anion of DCF interacted maybe with –OH and mainly with –NH_2_ groups of CS derivatives. Examining the –NH_2_ adsorption it can be seen that this was recorded at 3246 and 3251 cm^−1^ for CsSUC-DCF and CsMAL-DCF, respectively and at 3230, 3222 cm^−1^ for CsTACON-DCF and CsITA-DCF, respectively. It is clear that this shift is higher in derivatives that carboxylic monomer was polymerized as in the case of CsITA producing grafted macromolecular chains and also in CsTACON where two carboxyl groups are free, instead of the one that is in the cases of CsSUC and CsMAL. This is due to the higher extent and number of carboxyl groups in derivatives. In a similar way the –OH absorption was shifted too but in a smaller extent compared to –NH_2_ groups, from 3425 cm^−1^ to 3422 cm^−1^. Some intermolecular interactions can take place also between the carboxyl groups of DCF and these of derivatives. As can be seen the –COO^−^ absorbance was shifted from 1579 cm^−1^ to 1570–1575 cm^−1^. Concerning the supposed interactions between the secondary amino groups of DCF (>NH) and carboxyl groups of derivatives, these have not been proved from FTIR spectra due to the low-intensity absorption of >NH groups

Based on all of the above, it should be taken into consideration the pka of the materials and DCF. The pKa of DCF is 4.15. On the other hand, the pKa values of the carboxylic groups of the monomers added were found to be: 4.21 and 5.72 for SUC, 4.46 for TACON, 3.85 and 5.45 for ITA and 1.83 and 6.07 for MAL. The amino groups that are left unreacted, are indeed protonated under these acidic conditions and consequently, some repulsive forces may occur among them. However, it can be assumed that after the cross-linking process there are just a few residual NH_3_^+^ groups, therefore these repulsive forces are not significant and do not affect the adsorption.

**Figure 5:**
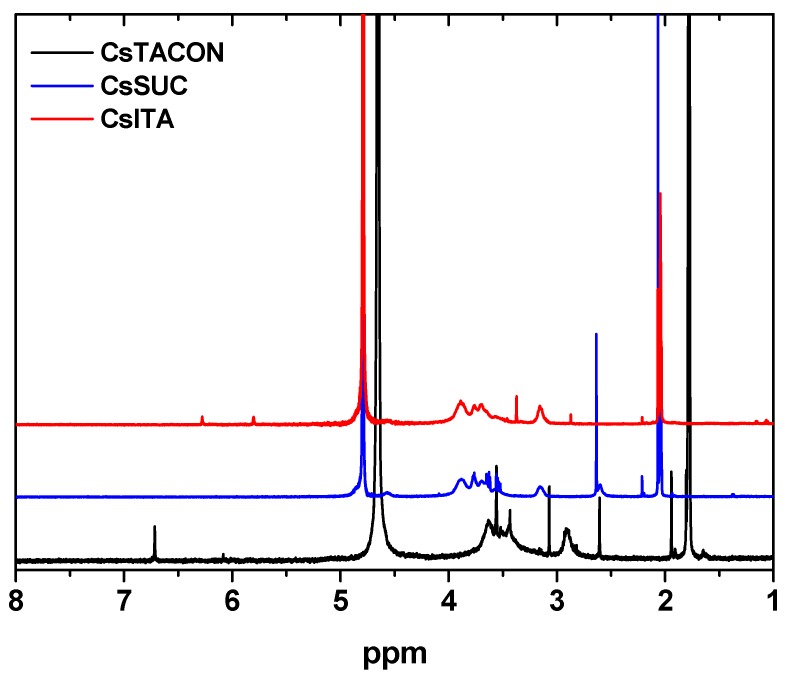
^1^H-NMR spectra of the synthesized chitosan derivatives.

^1^H-NMR spectroscopy was also adopted to confirm the successful grafting of chitosan. Figure 5 shows the recorded spectra of the three uncross-linked chitosan derivatives. In the case of CsITA, the *cis*- and *trans*-vinylic protons of itaconic acid can be clearly seen at δ 5.80 and 6.28 ppm, thus confirming the successful modification of chitosan.

Furthermore, depending on the carboxylic acid group that reacts, the methylene protons can be observed at 2.21 or 3.37 ppm. Therefore, we can undoubtedly say that chitosan has been modified with itaconic acid and that the double bond of itaconic acid has been preserved throughout the modification process. ^1^H-NMR (500 MHz, D_2_O-CD_3_COOD) δ (ppm): 2.07 (s, 0.3 H), 2.87 (s, 0.1) H), 3.16 (br, 1.0 H), 3.37 (s, 0.25 H), 3.56–3.89 (m, 6.8 H), 5.80 (s, 0.18 H), 6.28 (s, 0.18 H). In the case of CsTACON, the presence of *trans*-aconitic acid is evidenced by the peaks at 6.34 and 6.98 ppm, which correspond to the vinylic proton of *trans*-aconitic acid, depending on which carboxylic acid group reacts with chitosan. The CH_2_ group of *trans*-aconitic acid cannot be seen separately as the proton occurs among the chitosan protons, around 3.8 ppm. ^1^H NMR (500 MHz, D_2_O) δ (ppm): 2.06 (s, 0.04 H), 2.87 (s, 0.15 H), 3.17 (br, 0.8 H), 3.33 (s, 0.11 H), 3.53–3.89 (m, 4 H), 6.34 (s, 0.03 H), 6.98 (s, 0.11 H). The successful modification of chitosan with succinic anhydride (CsSUC) is evidenced by the peaks at 2.6 ppm, representing the two slightly differentiated CH_2_ groups from the succinic modification. ^1^H-NMR (500 MHz, D_2_O-CD_3_COOD) δ (ppm): 2.07 (s, 0.9 H), 2.60 and 2.63 (two peaks, 1.0 H), 3.15 (0.6 H), 3.52–3.88 (m, 5.6 H), 4.57 (br, 0.54H). Finally, after the reaction of chitosan with maleic anhydride, a new peak appears at 5.90 and 6.10–6.35 ppm (spectrum not shown here), which was assigned to –CH=CH–. It indicated the successful incorporation of the anhydride onto the chitosan backbone and the formation of CsMAL derivative.

**Figure 6:**
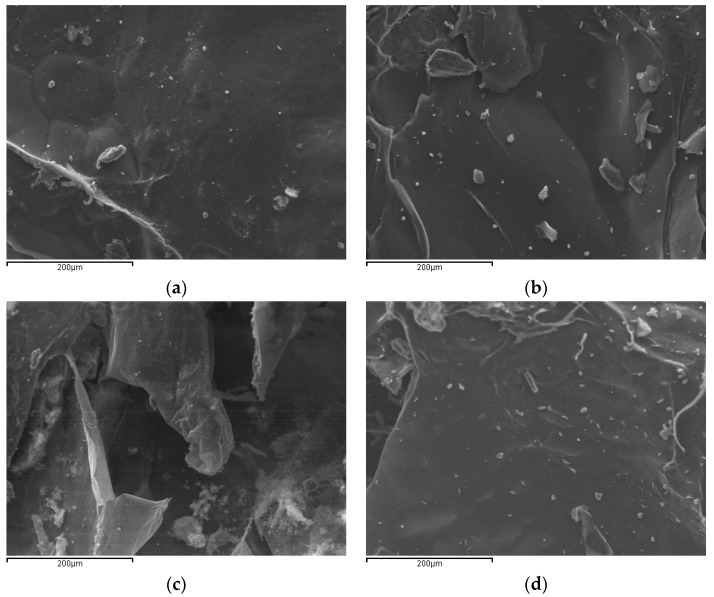
SEM images of the synthesized adsorbents: (**a**) CsSUC; (**b**) CsMAL; (**c**) CsITA; (**d**) CsTACON.

Another important characterization technique is scanning electron microscopy with which the surface morphology of the prepared materials can be evaluated. Figure 6 shows the SEM images of the prepared adsorbents.

All images exhibited a nonporous, nearly smooth membranous surface, occasionally containing a few random wavy points and microfibrils. Νo significant differences were observed due to the generally similar procedure during the synthesis for all materials (polymerization, washing/extraction, drying). Shape irregularities are mainly attributed to sample grinding and the different degree of grafting. Surface morphology is an important characteristic for the adsorption of several compounds. Smooth surfaces provide a wider area for the adsorption to take place. Since here there are no significant differences between the prepared derivatives, the adsorption of pharmaceutical compounds should be attributed to their different characteristics and mainly their different amount of reactive groups.

### 3.2. Adsorption Evaluation

#### 3.2.1. Effect of pH

**Figure 7:**
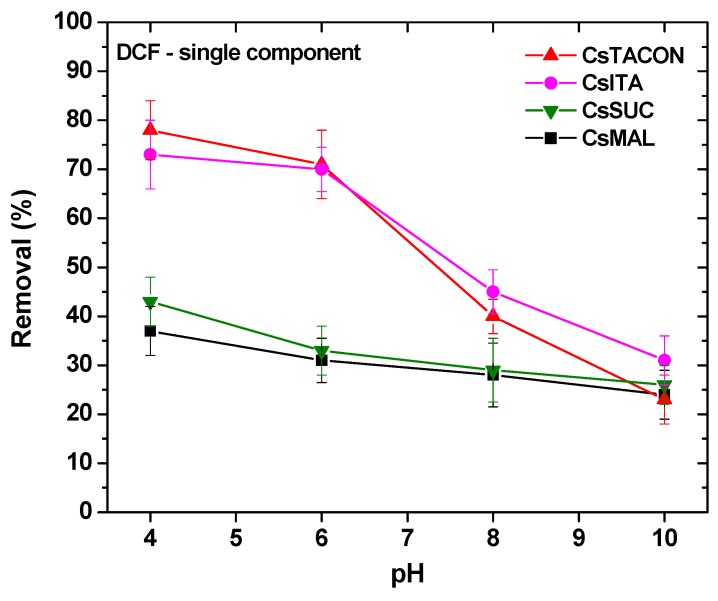
Effect of pH on the removal of DCF by chitosan adsorbents.

The majority of published papers related to adsorption begins with the presentation of pH-effect experiments. pH is one of the adsorption key-factors because the variation of this value can immediately and strongly change the whole adsorption behavior of the adsorbent [32,33,35,38,54,55,57]. In the present study, experiments were performed at pH = 4, 6, 8, 10 in order to check the sequential pH-behavior of the synthesized materials. The value of pH = 2 was omitted to avoid precipitation phenomena of the drug molecule (it was not in stable form). In addition, the value of pH = 12 was omitted, because chitosan materials presented difficulties to keep their rigidity and form. Figure 7 illustrates the pH-effect experiments in this first adsorption series.

As it can be observed for Figure 7, there are two clear trends in the synthesized materials. CsITA and CsTACON have the same pH-effect behavior presenting close quantitative DCF removals in the whole pH range. The optimum pH value found regarding DCF removal for all materials is pH = 4 (strong acidic pH-region). At this value, CsITA and CsTACON presented 73% and 78% removal of DCF, while the respective values for CsSUC and CsMAL were 43% and 38%. Increasing the solution from pH 4 to more neutral conditions (pH = 6 and 8), the differentiation of the prepared materials are clear. Although CsSUC and CsMAL presented removals of 33% and 31% at pH = 4, they reduced slightly this percentage to 29% and 28% at pH = 8, respectively; corresponded to only 3%–4% decrease. On the other hand, CsITA and CsTACON presented removals 70% and 71% at pH = 4, they reduced sharply this percentage to 45% and 40% at pH = 8, respectively; corresponded to 25%–30% decrease. Those materials (CsITA and CsTACON) continued this strong reduction at even alkaline conditions (pH = 10), where the removals were found to be 31% and 23%, respectively (meaning 15%–17% reduction). From all of the above, it is clear that CsSUC and CsMAL acted in a different way than CsITA and CsTACON. The latter may be due to the different structure and more especially to the different amount of carboxyl groups in each drug molecule. Based on Figure 1, the amino groups of DCF can be protonated at pH = 4 (–NH^+^), so this groups can easily attract the clear negatively carboxyl moieties (–COO^−^) of the chitosan adsorbents. Therefore, the difference in removals for CsSUC and CsMAL is negligible because both molecules have the same number of carboxyl groups (one), while CsITA (two carboxyl groups) and CsTACON (many carboxyl groups) have a different number of those groups. This may be the reason for which CsTACON presented sharp decrease with the increase of pH (increasing the pH. The amino group of DCF is deprotonated and the interaction with grafted carboxyl groups of chitosan becomes more difficult). All of the above will be verified later on using FTIR spectroscopy, by checking any shift of the spectral bands before and after drug adsorption.

#### 3.2.2. Effect of Initial Drug Concentration and Temperature

Although pH is the first and most important parameter in adsorption evaluation, another (even more quantitative) factor is the adsorption capacity of each material. This can be confirmed performing adsorption equilibrium experiments, in which the varying parameter is the initial concentration of pollutant (DCF in this study). The equilibrium amount in the solid phase (*Q*_e_, mg g^−1^) is the quantitative parameter to evaluate, and it is calculated according to the following equation (where *C*_i_ and *C*_e_ (mg L^−1^) correspond to the initial and equilibrium drug concentration, respectively; V (L) to the volume of the aqueous solution, and m (g) to the mass of chitosan adsorbents used):

(2) $$Q_{e}=\frac{\left(C_{i}-C_{e}\right)V}{m}.$$

Apart from the above, it is mandatory to determine the maximum theoretical adsorption capacity (*Q*_m_) of each adsorbent. For this reason, Langmuir (Equation (3)) [59], Freundlich (Equation (4)) [60], and Langmuir-Freundlich (L-F) (Equation (5)) [61] isotherm equations were applied to the experimental equilibrium points to fit them.

(3) $$Q_{e}=\frac{Q_{m}K_{L}C_{e}}{1{+K}_{L}C_{e}}$$

(4) $$Q_{e}{=K}_{F}C_{e}{}^{1/n}$$

(5) $$Q_{e}=\frac{Q_{m}K_{\mathrm{LF}}{\left(C_{e}\right)}^{1/b}}{1{+K}_{\mathrm{LF}}{\left(C_{e}\right)}^{1/b}}$$

where *Q*_m_ (mg g^−1^) corresponds to the theoretical maximum adsorption capacity; *K*_L_ (L mg^−1^) is the Langmuir equilibrium constant; *K*_F_ (mg^1−1/*n*^ L^1/*n*^ g^−1^) is the Freundlich constant; n (dimensionless) is the Freundlich constant representing the adsorption intensity; *K*_LF_ (L mg^−1^)^1/b^ is the L-F constant; b (dimensionless) is the L-F heterogeneity constant.

**Figure 8:**
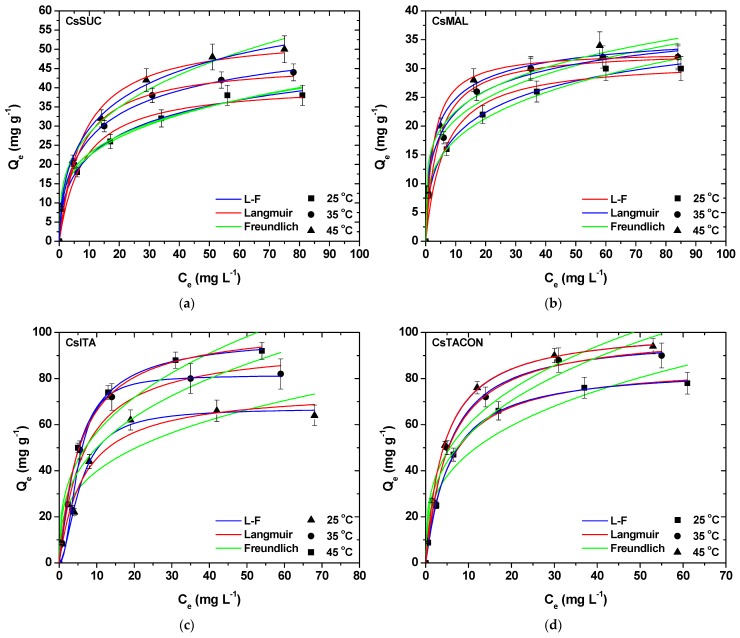
Isotherms for the removal of DCF by (**a**) CsSUC; (**b**) CsMAL; (**c**) CsITA; (**d**) CsTACON.

The adsorption equilibrium experimental points (25, 35, 45 °C), as well as the fitted isotherm curves, are presented in Figure 8a–d. As can be seen, by increasing the initial DCF concentration, the adsorption capacity in equilibrium (*Q*_e_) is also improved. The latter refers to all combinations of chitosan adsorbents and temperatures. Table 2 presents the fitting results of equilibrium data to the isotherm models.

A first comment is that L-F equation fitted better the equilibrium data for all combinations presenting the best correlation (0.983 < *R*^2^ < 0.999). Langmuir equation also fitted well the data, with slightly lower correlation coefficients though (0.957 < *R*^2^ < 0.998). Freundlich model was not able to satisfactorily fit in this study, presenting the lowest correlation (0.876 < *R*^2^ < 0.992). So, the adsorption evaluation regarding the maximum theoretical adsorption capacities (*Q*_m_) will be done for L-F parameters. The same findings can be confirmed from Table 3, based on error analysis (as described in Section 2.8.

The *Q*_m_ for CsTACON was higher than those of the other materials, at all temperatures studied. By altering the temperature from 25 to 35 °C, an increase (16%) of *Q*_m_ (from 84.56 to 98.34 mg g^−1^) was noted. Similar behavior was revealed after a further increase of temperature from 35 to 45 °C, improving by 5% (from 98.34 to 102.75 mg g^−1^). Analogous results were also observed for CsITA; an increase (21%) of *Q*_m_ (from 67.12 to 81.45 mg g^−1^) follows the increase of temperature from 25 to 35 °C, while a further improvement by 21% of *Q*_m_ value (from 81.45 to 98.94 mg g^−1^) reflects the temperature change from 35 to 45 °C. The interesting finding is that the increasing step between temperatures was 21% in both cases. In the case of other materials (CsSUC and CsMAL), the *Q*_m_ increase was nearly the same between temperatures. At 25 °C, the *Q*_m_ order is: CsTACON (84.56 mg g^−1^) > CsITA (67.12 mg g^−1^) > CsSUC (64.49 mg g^−1^) > CsMAL (47.07 mg g^−1^).

It is noteworthy to mention that some two-steps adsorption models that cover all cases of Langmuir, Freundlich and L-F sub-cases are given in the literature, but in the present work we use only the classic Langmuir, Freundlich and L-F models in order to use their constants in our proposed kinetic modelling.

#### 3.2.3. Effect of Contact Time—Kinetic Modeling

**Figure 9:**
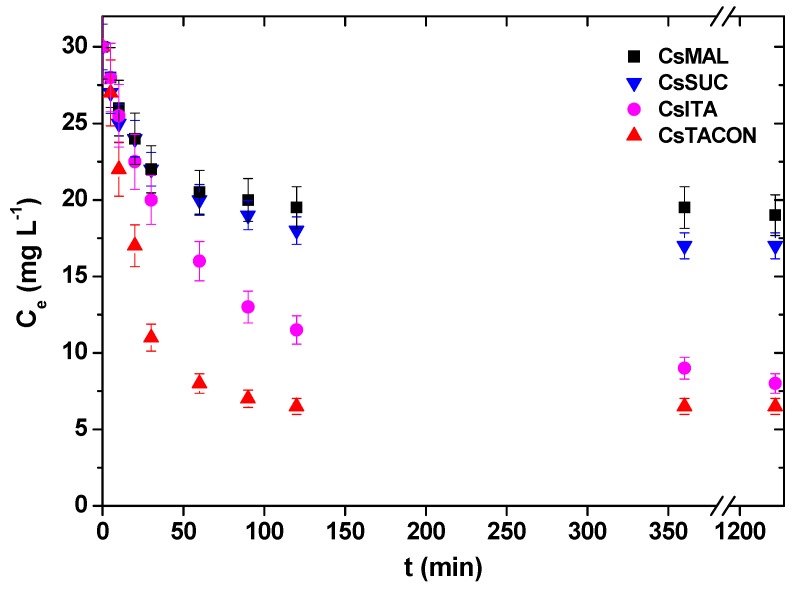
Experimental points (raw data) for the effect of contact time on the removal of DCF by chitosan adsorbents.

The evolution of bulk adsorbate concentration C for the four adsorbents used and for the temperature *T* = 25 °C, pH = 4, *N* = 150 rpm, *V* = 10 mL, *m* = 0.005 g and initial concentration *C*_i_ = 30 mg L^−1^ is shown in Figure 9. At a first glance, the kinetics is a rather regular one, the time scale is comparable between the adsorbents and the main difference is the final adsorbed amount which is completely compatible to the equilibrium experiment and the corresponding isotherms derived in the previous section.

The typical approach of describing the kinetic data by purely empirical models as the pseudo-first, pseudo-second order and power-law ones do not offer an inside to the process since the derived parameters are no physical parameters but depend on the experimental conditions [62]. On the other hand, phenomenological models are quite useful not only to provide information on adsorption mechanism and on adsorbent particle structure but also allows derivations of relations of general validity appropriate for process design.

The adsorption process consists of several steps: The first step is the convective particle diffusion of the solute from the liquid to the adsorbent solid surface. The second step is the incorporation of the solute on the adsorbent lattice. This step is considered to be very fast so the solute in the liquid and in the solid (adsorbed) state are always in local equilibrium. The third step is the diffusion of the solute to the interior of the adsorbent particle. The first step contribution to the kinetics can be eliminated with properly designed experiments. Intensive mixing (like one of the present experiments) confirms that external mass transfer resistance is negligible with respect to the internal one so it can be neglected. A surface diffusion process is considered i.e., diffusion occurring among the adsorption sites [33]. The diffusion process is dictated by the partial differential equation of transient diffusion for the adsorbed solute profiles in the adsorbent particle [63]. Hopefully, a simplified approach based on the assumption of a parabolic concentration profile and called Linear Driving Formula (LDF) approach can be utilized to replace the partial differential equation with an ordinary one [64].

Let us say that the instantaneous solute concentration in the bulk liquid is denoted as *C* and the instantaneous adsorbed solute to adsorbent mass ratio is denoted as *Q*. The motive force for adsorption is the difference between the actual adsorbed amount *Q* and the one in equilibrium with *C* (let us call it *Q*_e_). The governing equation for the evolution of *Q* assuming surface diffusion and employing LDF is:

(6) $$\frac{dQ}{dt}=K(Q_{e}-Q)$$

where *t* is time, *Q*_e_ the adsorbed material amount in equilibrium with bulk concentration *C*. The relation between *Q*_e_ and C can be found from the corresponding isotherms. From the results of the previous section, it is concluded that the Langmuir-Freundlich isotherm more appropriately describes the equilibrium experimental data

(7) $$Q_{e}=\frac{Q_{m}K_{\mathrm{LF}}{\left(C\right)}^{1/b}}{1{+K}_{\mathrm{LF}}{\left(C\right)}^{1/b}}.$$

The kinetic constant *K* is related to the diffusion coefficient of the solution in the adsorbent particle as *K* = 15*D*/*R*^2^ according to the LDF approximation (where R is the particle radius). The surface diffusion coefficient is generally dependent on the adsorbed amount *Q* [65], and this particular dependence is best described by Equation (8) (*D*_0_ is the diffusion coefficient for unloaded adsorbent):

(8) $$D=\frac{D_{0}}{1+KQ}$$

The mathematical problem is closed using the solute mass balance:

(9) $${C=C}_{0}-\frac{Q_{m}}{V}.$$

**Figure 10:**
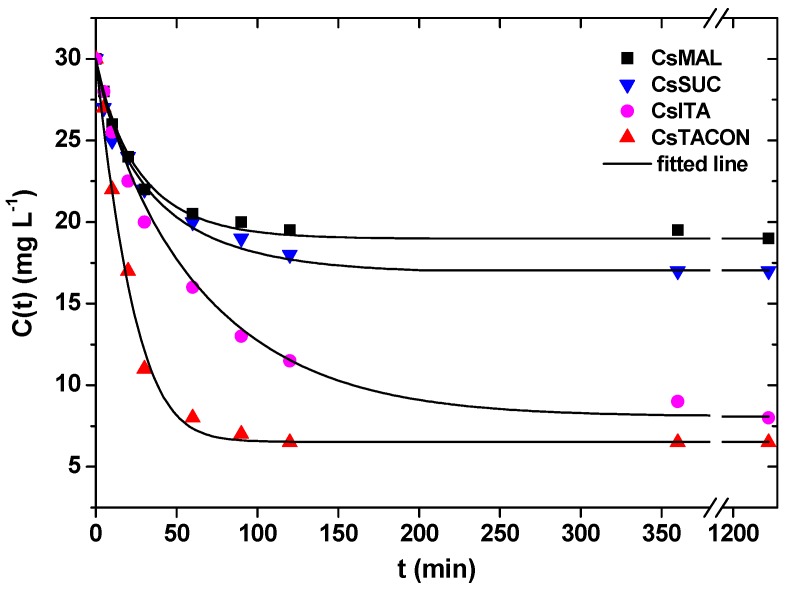
Comparison between experimental points (symbols) and model (lines) evolution curves for bulk solute concentration C(t).

The system of Equations (6)–(9) is solved numerically with initial value *Q*(0) = 0 using the Euler method to construct the curve *C*(*t*). The *D*_0_ and *k* values that lead to best fit (in the least square sense) to the experimental kinetics data are found through a minimization procedure. It is noted that the *Q*_m_, *K*_LF_ and *b* values used to fit each kinetic experiment are taken from Table 2. The use of the constant k in the model is essential since it alters the kinetic process by delaying it as it proceeds. Without this constant, the fitting to the experimental data would be no possible. The model kinetic curves resulted from the fitting procedure are shown in Figure 10. Assuming an average particle radius of 50 μm from the sieving process, the following values for the parameters *D*_0_ and *k* results in some basic points:

(i) The initial diffusion coefficient *D*_0_ increases in the following order: 0.39 × 10^−13^ m^2^ s^−1^ for CsITA, 0.7 × 10^−13^ m^2^ s^−1^ for CsTACON, 0.95 × 10^−13^ m^2^ s^−1^ for CsSUC and 1 × 10^−13^ m^2^ s^−1^ for CsMAL. The coefficient *D*_0_ is somewhat smaller for CsITA, increases for CsTACON and further increases from the rest two materials. This coefficient expresses the mobility of solute in the unloaded particle. On the other hand, the coefficient k denotes the (adverse) effect of already adsorbed material on the solute mobility in the particle.
(ii) The values of *k* are 0 for CsTACON, 0.02 g mg^−1^ for CsMAL, 0.03 g mg^−1^ for CsITA and 0.05 g mg^−1^ for CsSUC. The effect of adsorbed material on solute mobility varies from zero for CsTACON to a maximum for CsSUC. The particular ordering of the material with respect to *D*_0_ and *k* is related to their chemical and spatial structure and to the effect of the adsorbate (DCF) on this structure. The derived adsorption kinetic model can be used for the design of adsorption process (batch or continuous) using the particular adsorbents.

#### 3.2.4. Thermodynamic Evaluation

When the adsorption experiments include the study of temperature-effect, it is good to thermodynamically evaluate the process calculating some important parameters as enthalpy, entropy and free energy. Therefore, a thermodynamic analysis was also done based on the change of Gibbs free energy (*ΔG*^0^, kJ mol^−1^), change of enthalpy (*ΔH*^0^, kJ mol^−1^) and entropy change (*ΔS*^0^, kJ mol^−1^ K^−1^). The following system of equations can be used to calculate the aforementioned thermodynamic parameters (where *C*_s_ (mg L^−1^) is the amount adsorbed on a solid at equilibrium and *R* (8.314 J mol^−1^ K^−1^) is the universal gas constant) [66]:

(10) $$K_{c}=\frac{C_{s}}{C_{e}}$$

(11) $${\Delta G}^{0}=-RT\ln\left(K_{c}\right)$$

(12) $${\Delta G}^{0}{=\Delta H}^{0}-\;T\;{\Delta S}^{0}$$

(13) $$\ln\left(K_{c}\right)=\left(-\frac{{\Delta H}^{0}}{R}\right)\frac{1}{T}+\frac{{\Delta S}^{0}}{R}.$$

*ΔG*^0^ was given from Equation (11), while *ΔH*^0^ and *ΔS*^0^ were given from the slop and intercept of the chart between ln(*K*_c_) versus 1/*T* (Equation (13)). These parameters (at pH = 4), at selected initial DCF concentrations (5, 50, 100 mg L^−1^) and all temperatures (25, 35, 45 °C), were given in Table 3.

Negative values of *ΔG*^0^ showed in special cases (for *C*_i_ = 5 mg L^−1^ in the case of all materials and for *C*_i_ = 50 mg L^−1^ in the case of CsITA, CsTACON). The negativity in *ΔG*^0^ implies the spontaneous adsorption of DCF onto the chitosan adsorbents, so this spontaneity is only for low and medium initial DCF concentrations. Furthermore, the lowest energy state is the target in all natural processes. Hence, in order for the occurring adsorption phenomena to be spontaneous, the energy at the end of the process should be less than that of the initial state. Therefore, in our case, the *ΔG*^0^ values were negative only in some cases and not in all. Moreover, the decrease in those values with an increase of temperatures (from 25 to 35 and to 45 °C), which was found for all materials-DCF systems, indicates that higher temperatures favor the adsorption process. The latter is clearly depicted in Table 4.

The positive values of *ΔH*^0^ (for all materials-DCF systems) indicated the endothermic character of the process. These values were found to be inversely proportional to DCF concentration. This is related to the fact that in an endothermic process, in order for the adsorbate species (the drug molecules here) to be adsorbed, they have to displace more than one water molecule. This results in the endothermicity of the process and *ΔH*^0^ would, therefore, be positive. The magnitude of *ΔH*^0^ might also provide an indication (but not a certainty) of the adsorption type.

*ΔS*^0^ values were also found positive (similarly for all materials-DCF systems), a fact that reflects the affinity of the adsorbent towards the adsorbate species. The positive values of *ΔS*^0^ suggested extensive randomness at the solid/solution interface with some structural disorders both in the adsorbate and the adsorbent. The displaced by the adsorbate (DCF) water molecules, which were either diffused or adsorbed into the adsorbent, gained a large amount of transitional entropy and allowed the randomness in the system. The positive *ΔS*^0^ values also correspond to an increase in the degree of freedom of the adsorbed molecules [38].

#### 3.2.5. Adsorption in Mixture

**Figure 11:**
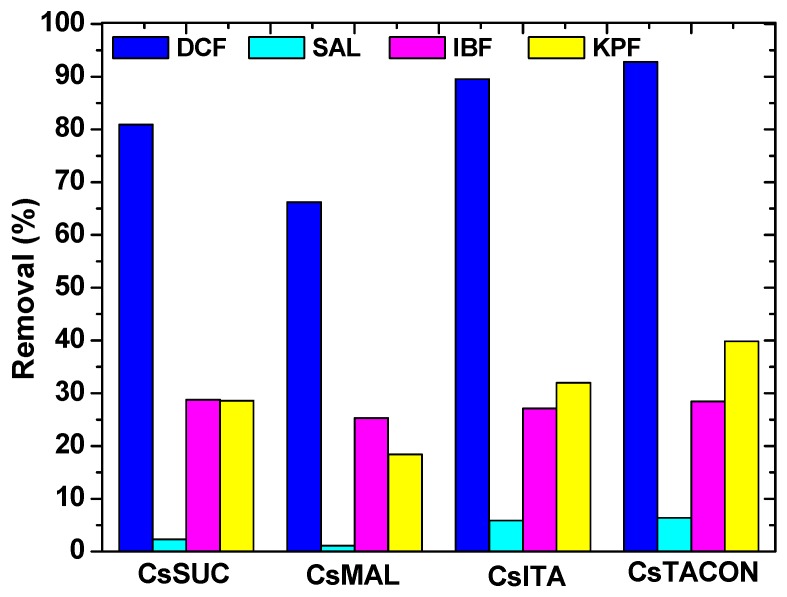
Adsorption of various drug compounds by chitosan adsorbents.

The ultimate target of any adsorption study is to check the ability of the adsorbent materials in the presence/co-existence other similar compounds. This is normal because in “real industrial life”, the wastewaters are not consisted only one pollutant each time, but a mixture of them. In the present study, some drug molecules were selected (of the same use: anti-inflammatory/analgesic), to check the adsorption behavior of the chitosan adsorbents prepared. In particular, the experimental conditions used were the optimum found from the single-component experiments As it can be observed from Figure 11, DCF presented the highest removal from the mixture CsTACON (92.8%) > CsITA (89.5%) > CsSUC (80.9%) > CsMAL (66.2%) compared to other pharmaceutical compounds. It is noteworthy that after DCF, KPF presented the next-higher removal. This may be attributed to the 2nd aromatic ring of the KPF molecule, which may interact with pi-pi interactions with the ring of DCF as it was confirmed in the literature [67]. From the other compounds, the removal order is IBF >> SAL which may due to the differentiation of their chemical structures. Another suggestion is the antagonistic movement of drug molecules to be adsorbed onto the active adsorption sites of the chitosan adsorbents. However, this cannot be easily evaluated or analyzed in real industrial samples, because some other additive reagents (during biological/chemical reactors of pharmaceutical industries), apart from the drug molecules, also exist. Another comment is that (unexpectedly) the chitosan grafted materials were found to be almost selective for DCF removal.

### 3.3. Desorption

**Figure 12:**
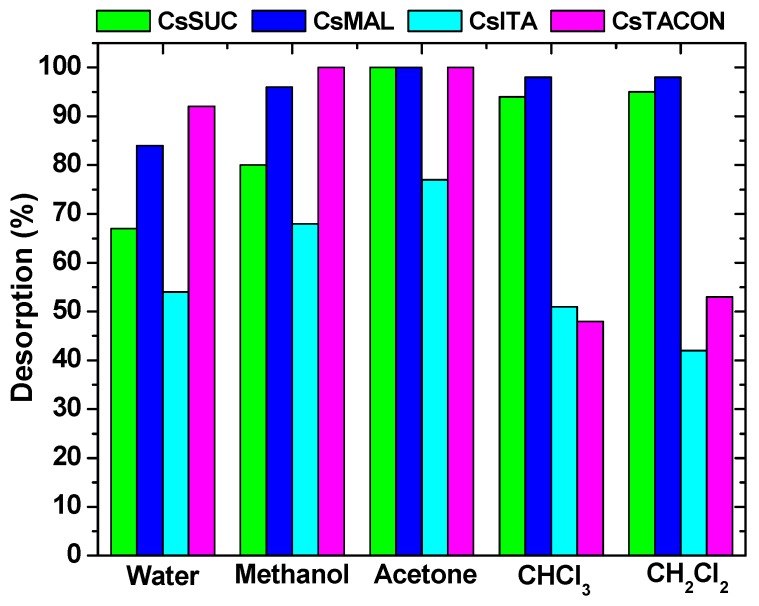
Desorption of various drug compounds by DCF-loaded chitosan adsorbents.

Another important parameter for evaluation is the ability of adsorbent materials to remove the adsorbed (molecule, ion) pollutant. Therefore the desorption study should be now analyzed. The selection of pollutants used for the present case is based on the polarity (organic solvents and deionized water) and environmental-friendly use (non-toxicity). It must be noted that the deionized water used as an eluent was previously adjusted to pH = 10 because the adsorption process was successfully achieved at acidic conditions (pH = 4), so in order to desorb, the conditions must be reversed. In Figure 12, the desorption percentage of the prepared materials is illustrated. The highest desorption ability was found for acetone (100% for CsTACON, CsSUC, CsMAL and 77% for CsITA) for all materials. On the contrary, it is interesting to mention that although CsTACON and CsITA presented the higher adsorption capacity, their desorption efficiency was not such high by classic organic solvents as dichloromethane and chloroform.

All of the above can be attributed to the combination of eluents and the nature of materials. To be clear, both adsorption and desorption processes are unique, because even with one change in adsorbent’s synthesis, a completely different adsorbent material is produced. Taking into account, the number of factors affecting the adsorption process (pH, contact time, temperature, agitation, salinity), the whole phenomenon can be easily characterized as multi-parametric. If anyone includes the supplementary process of desorption, which is taken place after adsorption, then the project is even more complexed and only scenarios can be hypothesized and not stated/confirmed explanations.

## 4. Conclusions

This study revealed the successful application of carboxyl-grafted chitosan materials as adsorbents for DCF removal. The optimum pH was 4; the amino groups of DCF can be protonated at pH = 4 (–NH^+^), so this groups can easily attract the clear negatively carboxyl moieties (–COO^−^) of the chitosan adsorbents. Regarding the adsorption capacity, CsTACON presented higher *Q*_m_ than those of the other materials, at all temperatures studied. By altering the temperature from 25 to 35 °C, an increase (16%) of *Q*_m_ (from 84.56 to 98.34 mg g^−1^) was noted, while similar behavior was revealed after a further increase of temperature from 35 to 45 °C, improving by 5% (from 98.34 to 102.75 mg g^−1^). A key-find in this study was the behavior of the prepared chitosan materials in simultaneously removing other compounds (salicylic acid, ibuprofen and ketoprofen). DCF presented the highest removal from the mixture in the order: CsTACON (92.8%) > CsITA (89.5%) > CsSUC (80.9%) > CsMAL (66.2%) compared to salicylic acid, ibuprofen and ketoprofen compounds. Desorption was achieved by using different eluants (either water or organic). The highest desorption ability was found for acetone (100% for CsTACON, CsSUC, CsMAL and 77% for CsITA) for all materials.

## Figures and Tables

**Table 1:** Adsorption experimental conditions for adsorption of DCF from aqueous media by chitosan derivatives.

	pH	*T*	*C*_i_	*N*	*t*	*V*	*m*
Experiment		(°C)	(mg L^−1^)	(rpm)	(min)	(mL)	(g)
Effect of pH	4, 6, 8, 10	25	30	150	1440	10	0.005
Effect of contact time	4	25	30	150	0–1440	10	0.005
Effect of initial drug concentration	4	25	5–100	150	30	10	0.005
Effect of temperature	4	25, 35, 45	5–100	150	30	10	0.005

**Table 2:** Equilibrium parameters for DCF adsorption from aqueous media onto chitosan derivatives at 25, 35, 45 °C (fitting to Langmuir, Freundlich and L-F equations).

Adsorbent		Langmuir Equation			Freundlich Equation			L-F Equation			
	*T*	*Q*_m_	*K*_L_	*R* ^2^	*K*_F_	*n*	*R* ^2^	*Q*_m_	*K*_LF_	*b*	*R* ^2^
	(°C)	(mg g^−1^)	(L mg^−1^)	(-)	mg^1−1/n^ L^1/n^ g^−1^	(-)	(-)	(mg g^−1^)	(L mg^−1^)^1/b^	(-)	(-)
CsSUC	25	41.18	0.127	0.971	11.58	3.135	0.992	64.49	0.158	1.922	0.994
	35	46.75	0.147	0.983	12.44	3.767	0.934	61.01	0.190	1.639	0.998
	45	54.20	0.129	0.977	13.67	3.195	0.988	82.22	0.159	1.852	0.996
CsMAL	25	31.48	0.161	0.969	9.35	3.631	0.988	47.07	0.197	1.964	0.996
	35	33.18	0.252	0.957	12.49	4.381	0.978	44.68	0.323	2.033	0.991
	45	33.08	0.398	0.972	13.93	4.772	0.962	38.31	0.445	1.630	0.990
CsITA	25	75.43	0.151	0.972	19.08	3.136	0.876	67.12	0.049	0.573	0.986
	35	94.93	0.155	0.967	22.84	2.942	0.883	81.45	0.033	0.467	0.983
	45	103.53	0.176	0.995	25.75	2.922	0.938	98.94	0.155	0.875	0.995
CsTACON	25	86.68	0.177	0.998	22.21	3.041	0.937	84.56	0.166	0.930	0.999
	35	100.84	0.184	0.998	25.32	2.929	0.938	98.34	0.175	0.934	0.997
	45	102.31	0.232	0.998	28.55	3.094	0.939	102.75	0.233	1.012	0.999

**Table 3:** Values of three different error analyses of isotherm models for adsorption of DCF onto chitosan derivatives.

Adsorbent		Langmuir Equation			Freundlich Equation			L-F Equation		
	T	SSE	SAE	ARE	SSE	SAE	ARE	SSE	SAE	ARE
	(°C)	(-)	(-)	(%)	(-)	(-)	(%)	(-)	(-)	(%)
CsSUC	25	31.69	10.75	12.48	13.44	7.94	6.23	5.18	4.30	2.88
	35	24.02	8.40	10.27	24.09	10.35	8.82	2.02	3.23	2.39
	45	44.44	12.31	13.91	22.44	10.45	7.28	5.71	5.77	3.82
CsMAL	25	20.17	9.12	11.38	7.71	5.93	5.62	2.10	2.67	2.03
	35	33.62	9.59	12.94	17.16	9.15	7.63	5.78	5.48	4.48
	45	22.75	9.40	10.19	32.01	11.92	10.63	6.62	5.67	3.72
CsITA	25	109.08	21.63	10.60	483.13	50.54	36.07	44.60	12.91	14.23
	35	198.61	27.15	13.14	701.31	60.68	40.34	81.88	17.14	18.63
	45	34.61	12.59	8.62	443.89	49.31	30.20	27.42	9.63	10.02
CsTACON	25	6.45	5.72	2.86	323.35	41.51	29.13	4.43	4.47	3.81
	35	16.79	9.11	3.88	426.33	49.29	31.97	14.44	9.15	5.13
	45	3.39	4.25	2.08	446.53	47.73	31.80	3.30	4.11	1.69

**Table 4:** Thermodynamic parameters for the adsorption of DCF onto chitosan grafted derivatives.

Adsorbent	*C*_i_	*T*	*Q*_e_	*K*_c_	*ΔG* ^0^	*ΔH* ^0^	*ΔS* ^0^
	(mg L^−1^)	K	mg g^−1^		kJ mol^−1^	kJ mol^−1^	kJ mol^−1^ K^−1^
CsSUC	5	298	8.42	5.25	−4.11	21.13	0.084
		308	8.61	6.14	−4.65		
		318	9.05	9.00	−5.81		
	50	298	32.04	0.47	1.87	17.01	0.051
		308	38.21	0.61	1.25		
		318	42.35	0.72	0.85		
	100	298	38.44	0.23	3.59	13.85	0.034
		308	44.02	0.28	3.24		
		318	50.04	0.33	2.90		
CsMAL	5	298	8.04	4.04	−3.43	32.28	0.121
		308	9.13	9.03	−5.63		
		318	9.16	9.04	−5.81		
	50	298	26.02	0.35	2.59	7.91	0.018
		308	30.04	0.43	2.17		
		318	30.21	0.44	2.24		
	100	298	30.04	0.18	4.30	5.87	0.005
		308	32.12	0.19	4.25		
		318	34.34	0.20	4.19		
CsITA	5	298	8.22	4.56	−3.76	36.32	0.134
		308	8.61	6.14	−4.65		
		318	9.24	11.50	−6.46		
	50	298	62.03	1.63	−1.21	22.06	0.079
		308	72.10	2.57	−2.42		
		318	74.02	2.85	−2.77		
	100	298	64.09	0.47	1.87	23.45	0.073
		308	82.08	0.69	0.93		
		318	92.04	0.85	0.42		
CsTACON	5	298	8.81	7.33	−4.94	17.70	0.076
		308	9.02	9.00	−5.63		
		318	9.23	11.50	−6.46		
	50	298	66.04	1.94	−1.64	19.30	0.070
		308	72.04	2.57	−2.42		
		318	76.52	3.17	−3.05		
	100	298	78.01	0.64	1.11	12.95	0.040
		308	90.32	0.82	0.51		
		318	94.21	0.89	0.32		
